# Intuitive vs Deliberative Approaches to Making Decisions About Life Support: A Randomized Clinical Trial

**DOI:** 10.1001/jamanetworkopen.2018.7851

**Published:** 2019-01-25

**Authors:** Emily B. Rubin, Anna E. Buehler, Elizabeth Cooney, Nicole B. Gabler, Adjoa A. Mante, Scott D. Halpern

**Affiliations:** 1 Division of Pulmonary and Critical Care Medicine, Massachusetts General Hospital, Boston; 2 Fostering Improvement in End-of-Life Decision Science, University of Pennsylvania, Philadelphia; 3 University of California, San Diego, School of Medicine, La Jolla; 4 Leonard Davis Institute of Health Economics, The Wharton School, University of Pennsylvania, Philadelphia; 5 The Palliative and Advanced Illness Research Center, Perelman School of Medicine, University of Pennsylvania, Philadelphia; 6 Division of Pulmonary, Allergy and Critical Care Medicine, Hospital of the University of Pennsylvania, Philadelphia; 7 Department of Biostatistics, Epidemiology, and Informatics, University of Pennsylvania, Philadelphia; 8 Department of Medical Ethics and Health Policy, Perelman School of Medicine, University of Pennsylvania, Philadelphia

## Abstract

**Importance:**

Patients with serious illnesses are often encouraged to actively deliberate about the desirability of life support. Yet it is unknown whether deliberation changes the substance or quality of such decisions.

**Objective:**

To identify differences in decisions about life support interventions and goals of care made intuitively vs deliberatively by patients with serious illnesses.

**Design, Setting, and Participants:**

Randomized clinical trial in which patients were asked to express treatment preferences in a series of clinical scenarios. Participants were 199 hospitalized patients aged 60 years and older with serious oncologic, cardiac, and pulmonary illnesses treated in a large, urban academic hospital from July 1, 2015, through March 15, 2016.

**Interventions:**

Patients in the intuitive group were subjected to a cognitive load and instructed to answer each question immediately based on gut instinct. Patients in the deliberative group were not cognitively loaded, were instructed to think carefully about their answers, and were required to explain their answers.

**Main Outcomes and Measures:**

Choices regarding life support (4 scenarios) and goals of care (1 scenario), concordance of these choices with patients’ valuations of health states that could follow from them, and decisional uncertainty.

**Results:**

Of 199 patients, 132 (66%) were male and the mean (SD) age was 67.2 (5.0) years. Similar proportions of patients in the intuitive group (n = 97) and the deliberative group (n = 102) said they would accept a feeding tube for chronic aspiration (42% vs 44%, respectively; difference, −2%; 95% CI, −16% to 12%; *P* = .79), antibiotics for life-threatening infection in the event of terminal illness (39% vs 43%, respectively; difference, −4%; 95% CI, −18% to 10%; *P* = .57), a trial of mechanical ventilation (59% vs 60%, respectively; difference,−1%; 95% CI, −15% to 13%; *P* = .88), and a tracheostomy tube (37% vs 41%, respectively; difference, −4%; 95% CI, −22% to 13%; *P* = .64). Patients in the deliberative group were slightly more likely than patients in the intuitive group to choose a palliative approach to treatment in the event of serious illness (45% vs 30%, respectively; difference, 15%; 95% CI, 1%-29%; *P* = .04). Across scenarios, decisional uncertainty was similar between the 2 groups (all *P* > .05), and intuitive decisions were either equally or more closely aligned with patients’ health state valuations than deliberative decisions.

**Conclusions and Relevance:**

In this study, encouraging hospitalized patients with serious illnesses to deliberate on end-of-life decisions did not change the content or improve the quality of these decisions. It is important to evaluate whether decision aids and structured communication interventions improve seriously ill patients’ choices.

**Trial Registration:**

ClinicalTrials.gov Identifier: NCT02487810

## Key Points

**Question:**

Do seriously ill patients’ decisions about life support interventions differ when made intuitively vs deliberatively?

**Findings:**

In a randomized clinical trial of 199 hospitalized patients aged 60 years and older with serious oncologic, cardiac, and pulmonary illnesses, encouraging patients to deliberate on end-of-life decisions did not change the content or improve the quality of those decisions.

**Meaning:**

It is important to evaluate whether commonly advocated decision aids and structured communication interventions improve seriously ill patients’ choices.

## Teaser

This randomized clinical trial compares decision making about life support interventions between a group of patients instructed to decide intuitively and a group instructed to decide deliberatively.

## Introduction

Patients with serious illnesses are often asked to articulate their preferences regarding treatment at the end of life. Such decisions include preferences for receiving specific medical interventions such as mechanical ventilation, and more general preferences for care designed to maximize longevity or comfort if these goals were to conflict.^1,2^ The widespread promotion of shared decision making and burgeoning development of formal decision aids represent efforts to foster careful deliberation about such decisions.^3,4,5,6^ The implicit assumption is that active deliberation will help patients choose care that is most consistent with their underlying values.

This norm of promoting deliberation is also supported by basic research showing that human cognition involves 2 different but interrelated modes of processing: one controlled by an intuitive system, which is fast and associative, and the other controlled by a deliberative system, which is slower, rule based, and analytic.^7,8,9^ A dominant view holds that the intuitive mode relies on multiple heuristics that can lead to systemic errors in judgment,^10^ whereas the deliberative system is commonly thought to help prevent such errors.^8^

However, a contrasting view holds that decisions made automatically can more faithfully integrate existing values and lead to normatively optimal judgments, particularly when making complex decisions.^11,12,13^ Other work has suggested that complex decisions are neither improved nor worsened by responding immediately^14,15^ and that deliberation may serve primarily to justify people’s original beliefs (a form of confirmation bias).^16^ A final view is that the mode of processing that will optimize any given decision depends on how closely the mode of thought matches the demands and features of the task.^17^

Although the mode of processing has been shown to influence peoples’ moral reasoning,^18,19^ depth of religiosity,^20^ exercise of self-control,^21^ and inclinations toward social cooperation,^22,23^ it is uncertain which mode ought to be promoted when engaging patients in making choices about end-of-life treatments.^24^ To inform the common practice of asking patients to deliberate about desired end-of-life treatment intensity, we conducted a randomized clinical trial in which we required hospitalized patients with serious illnesses to respond intuitively or deliberatively to a series of decisions regarding life support and general goals of care. We sought to identify differences in the aggressiveness of care chosen intuitively vs deliberatively and to determine which decision-making approach yielded choices that were more concordant with patients’ underlying valuations of health states that could foreseeably follow from those choices.

## Methods

### Patient Recruitment

**Figure 1.**
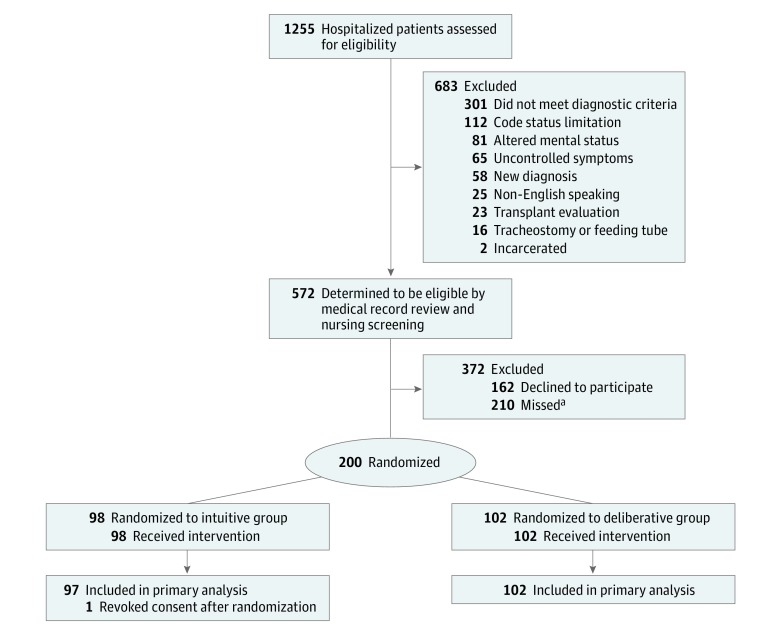
CONSORT Flow Diagram of Recruitment, Enrollment, and Data Analysis ^a^Missed patients were those who met eligibility criteria but were discharged from the hospital before they could be approached for consent.

We recruited patients who were hospitalized at the Hospital of the University of Pennsylvania from July 1, 2015, through March 15, 2016 (Figure 1). The trial protocol is included in Supplement 1. Eligible patients were aged 60 years or older and had advanced stage solid malignant neoplasm, advanced stage lymphoma, acute myeloid leukemia, New York Heart Association class III or IV congestive heart failure, or severe obstructive or restrictive lung disease. Patients with earlier-stage malignant neoplasms or class II heart failure were also eligible if they had been hospitalized previously that year (eFigure 1 and eFigure 2 in Supplement 2).

One of us (E.B.R.) reviewed the electronic medical record to identify eligible patients, and 2 others (A.E.B. and A.A.M.) screened the patient’s primary nurse to verify that patients had no exclusion criteria. The latter investigators then approached patients for written informed consent and conducted interviews using a standardized survey instrument on an electronic tablet that we had previously pilot tested with 20 patients to ensure facile comprehension. The University of Pennsylvania institutional review board approved this study. This study followed the Consolidated Standards of Reporting Trials (CONSORT) reporting guideline.

### Intervention

Just prior to starting the interview, consenting patients were randomized using the Qualtrics Randomizer tool to either an intuitive or deliberative decision-making group. Prior to beginning the interview, patients in the intuitive group were instructed to answer each of 5 questions about treatment preferences in different clinical scenarios immediately based on instinct (Table 1). Prior to each scenario, these patients were given a new 5-digit number and were instructed to remember it while hearing the scenario and to repeat it after answering each question. Imposing a cognitive load by asking people to focus on such a secondary task is commonly used in psychology to limit deliberation by occupying working memory.^25,26,27^

**Table 1.** Instructions Provided to Patients in the Intuitive and Deliberative Groups

Intuitive Group	Deliberative Group
It is very important that you answer each question immediately based on your gut instinct, giving the first answer that comes into your head.	It is very important that you take your time in thinking about these scenarios and consider each situation very carefully before you answer. You can take as much time as you need, but I want you to wait at least 1 full minute after I read each scenario before giving me your answer. Try not to let your gut feelings and emotions get in the way of making the best possible decision.
Before I describe each scenario, I am going to give you a string of 5 numbers. I am going to ask you to keep that string of numbers in your head while you are considering the scenario and making your decision. After you have made your decision for that scenario, I will ask you to try to repeat those numbers back to me.	While I ask you these questions, I am going to ask you to remember a single-digit number. When I am done with each question, I will ask if you remember the number.
The most important thing is that you try as hard as possible to keep the string of numbers in mind while you are hearing and answering the question. And remember to answer each question immediately with the first answer that comes into your head.	After you have answered each question, I will ask you to say a couple of sentences about why you made the decision you did.

Patients in the deliberative group were instructed to carefully consider all of their options and to wait at least 1 minute after hearing each scenario before answering the accompanying question (Table 1). They were told that they would be asked to explain why they answered as they did and were prompted for such explanations following each question. To promote structural balance of the interviews across groups without cognitively loading patients in the deliberative group, these patients were asked to remember 1 single-digit number throughout the entire exercise and to state that number following their answer to each question.

### Data Collection

Prior to hearing the clinical scenarios, and under the conditions for their assigned group described previously, all patients were presented with a single syllogistic reasoning task that had a logically valid but intuitively implausible conclusion (Premise 1: All mammals walk. Premise 2: Whales are mammals. Conclusion: Whales walk.). Patients were asked whether the conclusion followed logically from the 2 premises (the correct answer being no). Syllogistic reasoning tasks are commonly used to test whether interventions intended to promote intuitive responses were effective, as evidenced by more incorrect responses.^20,28^

Patients were given basic educational information about feeding tubes, endotracheal intubation, and tracheostomy. They were then presented, under the conditions for their assigned group, with 4 scenarios involving specific interventions: a feeding tube for chronic aspiration when there was a likelihood of dying sooner without the feeding tube; antibiotics for serious infection with life expectancy of a few months or likelihood of dying within 1 to 2 weeks without antibiotics; intubation for respiratory failure with estimated 50% chance of survival or certain death without intervention; and tracheostomy for prolonged respiratory failure or certain death without intervention (eFigure 3 in Supplement 2). The antibiotics and tracheostomy scenarios specified that, if the patient survived, he or she would live in a nursing home or other medical facility and would be dependent on others for activities of daily living.

After the first 20 patients had completed the study without demonstrating decisional fatigue, we added a fifth scenario in which patients were asked to indicate a preferred general approach to treatment in the event of critical illness: low intensity with focus on symptom management; moderate intensity with initial trial of aggressive care including life support, but no prolonged life support; or high intensity with all possible measures to prolong life (eFigure 3 in Supplement 2).

After stating a preference regarding each intervention, patients indicated on a 5-point Likert scale how strongly they agreed with the 3 statements in the uncertainty subscale of the Decisional Conflict Scale: (1) I am clear about the best choice for me; (2) I feel sure about what to choose; and (3) this decision is easy for me to make.^29^ After responding to all the scenarios, patients were asked to rate on a scale from 0 to 10 how much mental effort it took for them to remember the numbers while answering the questions about medical treatment.^30,31^

Next, without any cognitive load or specific instructions about deliberation, patients were asked to rate various single-dimension states of physical and cognitive debility on a 5-point Likert scale (worse than death, neither better nor worse than death, little better than death, somewhat better than death, much better than death). The raw distributions of these responses have been reported previously.^32^ Many of the health states reflected foreseeable outcomes of 1 or more hypothetical scenarios examined in the present study, including being confined to bed, needing constant care, living in a nursing home, or relying on a feeding tube or breathing machine. This portion of the interview was also added after the first 20 patients completed the study.

Finally, patients provided demographic information and indicated whether they had a living will and whether they had 2 or more conversations in the last year regarding their end-of-life treatment preferences.

### Outcomes

The primary outcomes were patients’ decisions about life support and goals of care in the 5 scenarios. Prespecified secondary outcomes were (1) decisional uncertainty, defined by the uncertainty subscale of the Decisional Conflict Scale; and (2) value-concordant choices, defined as refusal of forms of life support in scenarios in which acceptance would result in health states rated by the patient as the same as or worse than death. We hypothesized that patients in the deliberative group would make choices that were more concordant with their evaluation of underlying health states.

### Statistical Analysis

We used χ^2^ tests to compare choices about life support and overall care goals between groups and analyzed decisional uncertainty using 2-sample *t* tests. A priori, we hypothesized that patients who endorsed having living wills or having had 2 or more conversations in the last year about end-of-life preferences may be less strongly influenced by the deliberation intervention because such patients may have previously deliberated on similar choices. Thus, we prespecified 2 tests for effect modification by examining statistical interactions between the intervention and either the existence of a living will or the endorsement of prior goals-of-care conversations. Statistical significance of all tests was defined as a 2-sided *P* value less than .05.

A Bayes factor, which is a statistical index that quantifies the evidence in favor of a null hypothesis as opposed to an alternative hypothesis,^33^ was calculated for each decision using R package BayesFactor. The Bayes factor as reported in R package BayesFactor is a ratio of the likelihood probability of the alternative hypothesis to the likelihood probability of the null hypothesis. A lower ratio therefore suggests a higher degree of evidence for the null hypothesis.

We used Stata statistical software version 13.1 (StataCorp) to calculate sample size. With 100 patients in each group, the study would have 80% power to detect differences of 20% above or below baseline rates of 50% in the proportions of patients choosing each form of life support or goal of care with a type I error rate of 0.05. Power to detect the same 20% absolute effects would increase as baseline rates deviated away from 50%. It is difficult to identify a minimal clinically important difference in rates of selecting certain treatments or goals. But we reasoned that such 20% absolute differences, if found, would convincingly suggest that the decision-making strategies produced different results. Smaller differences may also be clinically important, yet may go undetected owing to low statistical power. However, providing Bayes factors to accompany each result enables readers to gauge the risk of such type II errors.

## Results

### Trial Population

Among 362 eligible patients approached for this study, 200 (55%) consented and were randomized: 98 to the intuitive group and 102 to the deliberative group (Figure 1). All 200 patients completed the interview. One patient in the intuitive group withdrew consent after completing the interview and was excluded from analyses. Of 199 patients, 132 (66%) were male and the mean (SD) age was 67.2 (5.0) years. Patients’ baseline characteristics were similar in the 2 groups (Table 2).

**Table 2.** Baseline Patient Characteristics

Characteristic^a^	Patients, No. (%)		
	Total Sample (N = 199)	Intuitive Group (n = 97)	Deliberative Group (n = 102)
Demographic characteristics			
Age, mean (SD), y	67.2 (5.0)	66.5 (4.8)	67.9 (5.2)
Male	132 (66)	64 (66)	68 (67)
White	170 (85)	86 (89)	84 (82)
Married or partnered	140 (70)	69 (71)	71 (70)
Education >12 y	151 (76)	75 (77)	77 (76)
Annual income ≥$100 000	67 (39)	29 (30)	38 (37)
Christian	131 (66)	70 (72)	61 (60)
Medical history			
Advanced solid malignant neoplasm	29 (15)	14 (14)	15 (15)
Less advanced solid malignant neoplasm	3 (2)	2 (2)	1 (1)
Acute leukemia	38 (19)	19 (20)	19 (19)
Lymphoma	26 (13)	9 (9.3)	17 (17)
Multiple myeloma	21 (11)	14 (14)	7 (7)
Other hematologic malignant neoplasm	8 (4)	5 (5)	3 (3)
Severe lung disease	23 (12)	11 (11)	12 (12)
Congestive heart failure			
New York Heart Association class III or IV	48 (24)	23 (24)	25 (25)
New York Heart Association class II	3 (2)	0	3 (3)
Previous end-of-life discussions^b^	116 (57)	55 (57)	61 (60)
Living will	112 (56)	54 (56)	58 (57)
Current health rating fair or poor	116 (58)	57 (59)	59 (58)
Prior hospitalization in intensive care unit	118 (59)	58 (60)	60 (59)

^a^ All characteristics showed nonsignificant differences with *P* > .05.

^b^ Endorsed having had 2 or more conversations regarding end-of-life treatment preferences in the last year.

### Effectiveness of Intervention

The mean (SD) rating of mental effort was significantly higher in the intuitive group (5.5 [2.26] of 10) than in the deliberative group (0.23 [1.23] of 10) (difference, 5.27; 95% CI, 4.78 to 5.78; *P* < .001) (eFigure 4 in Supplement 2). Similarly, 0% of patients in the intuitive group rated the effort required for the number recall task as 0, whereas 93% of patients in the deliberative group did so (difference, −93%; 95% CI, −98% to −88%; *P* < .001). In the intuitive group, 57% of participants correctly answered the syllogism question, vs 66% in the deliberative group (difference, −9%; 95% CI, −22% to 6%; *P* = .14).

### Care Choices

**Figure 2.**
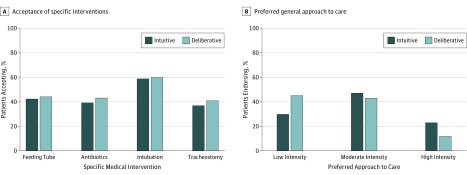
Acceptance of Specific Interventions and Preferred General Approach to Care Among Patients Making Decisions Intuitively and Deliberatively A, In the intuitive group, n = 97 for all interventions except tracheostomy and n = 57 for tracheostomy (only patients who stated they would accept intubation were asked whether they would accept tracheostomy). In the deliberative group, n = 102 for all interventions except tracheostomy and n = 61 for tracheostomy. B, In the intuitive group, n = 87. In the deliberative group, n = 93. *P* values were calculated using χ^2^ test.

Similar proportions of patients in the intuitive and deliberative groups said they would accept a feeding tube (42% vs 44%, respectively; difference, −2%; 95% CI, −16% to 12%; *P* = .79), antibiotics (39% vs 43%, respectively; difference, −4%; 95% CI, −18% to 10%; *P* = .57), intubation (59% vs 60%, respectively; difference,−1%; 95% CI, −15% to 13%; *P* = .88), and tracheostomy (37% vs 41%, respectively; difference, −4%; 95% CI, −22% to 13%; *P* = .64) (Figure 2A).

The Bayes factors calculated using R package BayesFactor were as follows: feeding tube, 0.1802; antibiotics, 0.2029; intubation, 0.1747; and tracheostomy, 0.2453. These Bayes factors suggest that there is moderate evidence in support of the null hypothesis that the interventions do not produce different distributions of choices.^33^

In the scenario regarding general goals of care, patients in the deliberative group were slightly more likely to prefer low-intensity approaches to care (45% vs 30%, respectively; difference, 15%; 95% CI, 1%-29%; *P* = .04) (Figure 2B). The Bayes factor was 0.9861, suggesting that there is anecdotal evidence for the null hypothesis that the interventions do not produce a different distribution of choices.

Patients who endorsed having living wills or having had 2 or more conversations in the last year about end-of-life preferences were generally less likely to choose aggressive approaches to care, but the corresponding interaction terms from regression models suggested that neither variable consistently modified the effect of the intuitive vs deliberative intervention (eTable 1 in Supplement 2).

### Decisional Uncertainty

Decisional uncertainty was low for all decisions and did not differ by group (eTable 2 in Supplement 2). On a scale from 0 (extremely certain about best choice) to 100 (extremely uncertain about best choice), mean (SD) uncertainty for the decision about tracheostomy was 29 (25) in the intuitive group and 24 (25) in the deliberative group (difference, 5; 95% CI, −3 to 15; *P* = .22). For all other scenarios, decisional uncertainty was lower, and the differences between the groups were smaller.

### Value-Concordant Care Choices

For nearly every combination of specific intervention choices and resulting health states, a smaller proportion of patients in the intuitive group than in the deliberative group accepted interventions that would leave them in states they valued as equal to or worse than death (Table 3), but none of these results were significant using a Fisher exact test. For example, among patients who stated that needing care all the time would be a state equal to or worse than death, 10% in the intuitive group and 32% in the deliberative group said they would accept a tracheostomy (difference, −22%; −45% to −0.003%; *P* = .08). The corresponding Bayes factor was 1.475, providing anecdotal evidence that intuitive decision making more commonly produces value-concordant choices. The remaining Bayes factors suggested either anecdotal or moderate evidence for the null hypothesis, and none provided evidence that deliberative decision making increased the frequency of value-concordant choices.

**Table 3.** Value-Discordant Decisions Among Patients Responding Intuitively or Deliberatively^a^

Decision	Intuitive Group, %	Deliberative Group, %	Difference (95% CI), %	*P* Value
Accept tracheostomy				
Bed bound equal to or worse than death (intuitive, n = 26; deliberative, n = 37)	15	24	−9 (−29 to 10)	.53
Need care all the time equal to or worse than death (intuitive, n = 21; deliberative, n = 25)	10	32	−22 (−45 to −0.003)	.08
Live in a nursing home equal to or worse than death (intuitive, n = 18; deliberative, n = 26)	22	19	3 (−21 to 27)	>.99
Rely on a breathing machine equal to or worse than death (intuitive, n = 25; deliberative, n = 32)	16	22	−6 (−26 to 14)	.74
Accept antibiotics				
Bed bound equal to or worse than death (intuitive, n = 50; deliberative, n = 68)	24	35	−11 (−28 to 5)	.23
Need care all the time equal to or worse than death (intuitive, n = 43; deliberative, n = 54)	21	35	−14 (−32 to 3)	.18
Live in a nursing home equal to or worse than death (intuitive, n = 33; deliberative, n = 46)	30	30	0 (−21 to 20)	>.99
Accept feeding tube				
Rely on a feeding tube equal to or worse than death (intuitive, n = 46; deliberative, n = 54)	15	30	−15 (−30 to 2)	.10

^a^ This table illustrates the proportion of patients in each group who stated they would accept a specific intervention that would result in 1 or more health states considered by the patient to be worse than death. Comparisons were conducted only when a scenario specifically provided that acceptance of the given intervention would result in a certain health state. *P* values were calculated using Fisher exact test.

## Discussion

The task of making decisions about the intensity of medical treatment one desires at the end of life involves integrating personal values regarding the relative importance of quality and quantity of life with information about the nature and efficacy of medical interventions. Given this complex interplay of factors, the relative importance of intuition and deliberation in making optimal decisions about medical interventions at the end of life is unclear. However, decision aids and other strategies designed to help patients deliberate on complex medical decisions are proliferating.^4,34^

This randomized clinical trial of intuitive vs deliberative approaches to choosing care options in states of advanced illness suggests that deliberation may be insufficient to improve choices about end-of-life care. Specifically, we found that encouraging hospitalized patients with a diverse array of serious illnesses to deliberate did not yield preferences regarding receipt of life-sustaining interventions across 4 clinical scenarios that differed from those revealed by patients who were forced to respond intuitively. Importantly, the patients we studied are precisely those who are likely to be encouraged to deliberate on such choices in the near term. Thus, these data extend the results of a prior study showing that deliberation also failed to change choices among healthy people asked to imagine being ill.^24^

There are several possible explanations for the similar preferences expressed by patients responding intuitively and deliberatively. First, it is possible that our interventions did not create adequate separation between how patients in the 2 groups made their decisions. It is unlikely that patients given the intuitive instructions could engage in significant deliberation because they were required to respond immediately. Furthermore, the enormous difference between groups in patients’ subjective ratings of mental effort suggests that patients in the intuitive group had more of their working memory occupied by the task, crowding out the capacity for deliberation.^30^ By contrast, patients in the deliberative group might have responded intuitively despite explicit instructions to deliberate. Similarly, a relatively brief period of deliberation in the context of an interview with a survey instrument may not yield different conclusions than intuitive decision making.

A second possible explanation for the similarity in the groups’ distributions of decisions about life support is that patients with serious illnesses may already have strong preferences regarding end-of-life care. If so, patients may be able to express their true preferences spontaneously, making deliberation unnecessary. The present data cannot disprove this explanation, but it runs counter to prior evidence. For example, the observations that default options in advance directives dramatically influence seriously ill patients’ preferences for life support suggest that many patients do not hold previously assembled preferences for such treatments.^35,36^

Third, deliberation may truly be ineffective as a means of influencing end-of-life decisions. We cannot be certain which of these mechanistic explanations is correct. However, all 3 possibilities yield the same conclusion that simply encouraging seriously ill patients to deliberate about specific decisions to receive mechanical ventilation, tracheostomy, feeding tubes, and antibiotics in states of advanced illness is unlikely to change their choices. Although failing to identify differences in the choices made does not necessarily mean that no differences exist, the Bayes factors reported in this article provide some assurance that the results do not represent type II errors.

An important strength of this study is that it also sought to determine whether deliberation improved the quality of patients’ decisions. First, we explored rates at which patients’ treatment decisions were concordant with their underlying valuations of resultant health states.^37,38^ Deliberation could improve rates of value-concordant choices even without altering the overall distributions of responses if it made some patients more likely to forgo interventions that would lead to intolerable health states and others more likely to accept interventions that would lead to tolerable states. However, we found no evidence of such benefits. Across all scenarios, patients in the intuitive group were as likely or more likely to appropriately forgo interventions that would foreseeably lead to health states they had personally valued as equal to or worse than death. Indeed, none of the Bayes factors suggested even weak evidence in favor of deliberation.

We also gauged decision quality by measuring patients’ uncertainty regarding decisions made intuitively or deliberatively. Deliberation has reduced decisional uncertainty in other contexts,^25,39,40^ although this may not reflect better decisions because the effect may be an artifact of confirmation bias.^41^ Regardless, we found similarly low levels of decisional uncertainty in both groups.

Although encouraging deliberation neither changed nor improved the quality of choices for specific life-sustaining interventions, there was a signal that patients in the deliberative group may have been more likely to choose a low-intensity overall approach to treatment. It is plausible that careful reflection may have helped patients articulate their broader preferences without influencing their preferences for specific treatments. However, even for this analysis the Bayes factor provided anecdotal support for the null hypothesis that the interventions do not produce a difference in distribution of choices. Furthermore, this was the only question that had 3 answer choices, and the choices were ordered from low to high intensity for all patients. Thus, patients who were cognitively loaded may have more commonly relied on heuristics such as extremeness aversion (tendency to pick middle options)^42,43^ and recency effects,^44,45,46^ both of which would bias toward more aggressive choices among patients responding intuitively.

### Limitations

The results of this study should be interpreted in light of several limitations. First, the patients were predominantly male, white, and married or partnered. These factors, combined with the fact that patients were recruited from a single center, may limit generalizability. Second, although more than half of the seriously ill patients we approached enrolled in this trial, it is possible that patients who declined enrollment may have had systematically different preferences for end-of-life treatment. Third, we encouraged patients to deliberate for as long as they wished, but we did not test a prolonged period of deliberation or “facilitated deliberation,” as is advocated under shared decision making. Thus, our results indicate that simply encouraging deliberation is unlikely to improve choices, but it is uncertain whether actively facilitating deliberation, including providing prognostic information and describing a range of potential outcomes, might influence choices. Likewise, it is uncertain whether and how the inclusion of family members or other close contacts in deliberation would influence choices.

Fourth, it is possible that participants misunderstood the scenarios presented, which could bias the results toward the null. However, we pilot tested the scenarios among 20 patients prior to launching the trial and observed good understanding. Fifth, more than half of the patients who did enroll in the study had engaged in prior deliberation about end-of-life treatments, as reflected by having a living will or having 2 or more conversations about desired end-of-life treatment in the last year (Table 2). We found no evidence that deliberation changed choices even among patients without these objective markers of prior deliberation, nor that prior deliberation modified the effects of the intervention. However, it is possible that many may have contemplated these choices previously without endorsing either objective marker of prior deliberation.

Sixth, the sample size may have limited the study’s ability to detect true effects. In an effort to quantify the strength of the evidence in favor of the null hypothesis (ie, that the interventions did not alter the distributions of choices made), we calculated Bayes factors for the 4 primary decisions made by the patients about specific interventions. These analyses provide moderate evidence in direct support of the null hypothesis.^33^

## Conclusions

In summary, this randomized clinical trial finds that encouraging seriously ill patients to deliberate before making choices regarding life support may not increase the likelihood that patients will make decisions that best promote their values. Although the scenarios were hypothetical, the findings are salient because the hospitalized patients participating in the study were at high risk of encountering the decisions and health states being studied. These findings may not generalize to other types of patients or decisions, or to contexts in which clinicians guide patients through a deliberative process. However, the results highlight the importance of testing the effects of deliberation on choices made in these other contexts, rather than assuming that deliberative approaches will necessarily improve patients’ decision-making processes.

## Notes

Supplement 1. **Trial Protocol**

Supplement 2. **eFigure 1.** Study Inclusion and Exclusion Criteria **eFigure 2.** Distribution of Primary Diagnoses Among Patients Enrolled in the Study **eFigure 3.** Scenarios Requiring Decision About Life-Sustaining Treatment **eFigure 4.** Distribution of Subjective Rating of Mental Effort in Intuitive Arm Versus Deliberative Arm **eTable 1.** Interaction Between Selected Characteristics and Treatment Arm for the Outcome of Treatment Decisions **eTable 2.** Decisional Uncertainty by Arm

Supplement 3. **Data Sharing Statement**
