# Supplementary material for: Intuitive vs Deliberative Approaches to Making Decisions About Life Support: A Randomized Clinical Trial
Source: JAMA Netw Open. 2019 Jan 25;2(1):e187851. doi: 10.1001/jamanetworkopen.2018.7851 (PMC6484534; doi:10.1001/jamanetworkopen.2018.7851)
Supplement: Supplement 1. — Trial Protocol [file jamanetwopen-2-e187851-s001.pdf]

Protocol Title:	Intuition Versus Deliberation in Decisions About Life-Sustaining Medical Therapies
Short Title:	Intuition Versus Deliberation
Investigators:	Emily B. Rubin, MD, JD<br>Scott D. Halpern, MD, PhD
Clinical Trials ID:	NCT02487810
Protocol Description:	The purpose of this study is to determine whether there are systematic differences between the decisions patients make intuitively versus deliberatively about life-sustaining medical therapies. The targeted population is inpatients at the Hospital of the University of Pennsylvania with serious medical problems. The study will involve facilitated interviews with patients using a survey instrument developed in Qualtrics.

Table of Contents

1 Protocol 3
1.1 Objectives 3
1.2 Background 3
1.3 Study design 3
1.3.1 Design 3
1.3.2 Study duration 3
1.3.3 Target population 3
1.3.4 Group modifications 4
1.3.5 Method for assigning subjects to groups 4
1.4 Study instruments 4
1.5 Subject recruitment 5
1.6 Subject compensation 5
1.7 Study Procedures 6
1.7.1 Consent 6
1.7.2 Procedures 6
1.7.3 Administration of surveys and/or process 7
1.8 Human research protection 8
1.8.1 Data management 8
1.8.2 Subject confidentiality 8
1.9 Risk/Benefit 8
1.9.1 Potential study risks 8
1.9.2 Potential study benefits 9
1.9.3 Risk/benefit assessment 9
1.10 Summary of changes to protocol 9
2 Statistical analytic plan 10
2.1 Summary of changes to statistical analytic plan 10
3 References 11

1 Protocol

1.1 Objectives

Aim 1: Determine whether there are systematic differences between the decisions patients make intuitively versus deliberatively about life-sustaining medical interventions.

Aim 2: Evaluate how decisions about life-sustaining treatments reached intuitively and those reached deliberatively map with the stated underlying values of the patient.

1.2 Background

Research in cognitive psychology has demonstrated convincingly that human cognition involves two different but interrelated modes of processing. The first, which is fast and associative, has been attributed to an experiential or intuitive system (system 1); the second, which is slower, rule-based and analytic, has been attributed to a rational or deliberative system (system 2)[1-3]. It is widely accepted that the intuitive process involves multiple heuristics that can lead to biases, which in turn can lead to serious systemic errors in judgment[4]. The deliberative system is generally thought to help serve as a check against such errors[2]. A minority view, however, holds that decisions made under automatic or unconscious conditions will more faithfully integrate existing values and can lead to normatively optimal judgments, particularly where the decisions in question are complex, requiring the organization and synthesis of several factors[5-6]. The mode of processing used by an individual has been shown to impact such outcomes as the use of deontological versus utilitarian moral reasoning[7-8], depth of religiosity[9], and inclination toward social cooperation[10-11]. There has been a strong movement in health care towards the development of decision aids that foster deliberation[12], but we know little about the use and relative merits of intuitive and deliberative thinking in decisions regarding life-sustaining medical interventions. Patients and surrogates are asked to make judgments about the desirability of initiating or continuing such treatments under widely disparate circumstances: on admission to the hospital, in the context of prolonged discussions over multiple visits with outpatient providers, and in time-sensitive emergency situations. Treatment preferences of ill patients[13], consistency of delivered care with patient preferences, and the utility of advance care planning in improving such consistency[14] have all been extensively researched. However, there is no empirical evidence whether the mode of cognitive processing employed by patients systematically influences the decisions they reach about the desirability of life-sustaining medical treatments.

1.3 Study design

1.3.1 Design

Pilot prospective randomized trial.

1.3.2 Study duration

Subjects will be accrued over a period of six months starting in June 2015. The total time it will take the research coordinator to explain the study, obtain consent and for a subject to complete the two sessions will, conservatively, take no more than 45 minutes.

1.3.3 Target population

We will be recruiting 200 patients aged 60 or older with chronic respiratory, oncological or cardiovascular diseases. This sample size would provide at least 80% power to detect differences of 20% above or below baseline rates of 50% in the proportions of patients choosing each form of life support

or goal of care with a type I error rate of 0.05. Power to detect the same 20% absolute effects would increase if baseline rates deviate away from 50%.

1.3.4 Group modifications

Subjects enrolled in this trial will be randomized into two groups, an intuitive arm and a deliberative arm. Patients in each arm will be asked all of the same questions. The only difference between the arms will be the instructions regarding how and when to answer the series of hypothetical questions regarding medical interventions. The instructions will be designed to influence patients to think either intuitively or deliberatively about the questions regarding life-sustaining interventions. We will be pilot testing both the deliberative and the intuitive arm prior to starting formal recruitment. The details of the pilot testing are discussed in the Study Procedures section of this proposal. Once we have determined the optimal method, we will submit an IRB modification that reflects those methods

1.3.5 Method for assigning subjects to groups

Consenting subjects will be randomized with a 50% probability to each trial arm (intuitive versus deliberative procedures) using Qualtrics. The research coordinator/PI will become unblinded to the patients allocation when they open the electronic survey

1.4 Study instruments

Demographics survey:
The demographics survey will consist of basic information such as race, education, and religion. We will also ask questions about prior experience with intensive care and whether the patient has a document outlining preferences for care at the end of life.

Question regarding general treatment preference:
We will ask a single general question regarding the patient's treatment priorities in the event of serious illness. The response to this question is modeled on one used in a Study to Understand Prognoses and Preferences for Outcomes and Risks of Treatments (SUPPORT) study[15]. The question acknowledges that in general, most people wish to both live as long as possible and avoid pain and suffering, but that in some situations choosing between these two goals may be necessary. It then asks patients, if they are in a situation where such a choice is needed, if they would they desire a plan of care that focuses on extending life as much as possible, even if it means having more pain and suffering, or a plan of care that focuses on relieving pain and suffering, even if that means not living as long.

Health state valuation questions:
We will ask a series of questions designed to understand how the patient values various health states. We will use three distinct health states: the patient's current state of health and two hypothetical health states that span four domains - cognitive functioning, physical mobility, self-care and pain/discomfort. We will first ask the patient to rate each state of health on a 10-point Likert scale with 0 being much worse than death and 10 being much better than death[16]. We also will ask patients to evaluate a variety of single item health states on a visual analog scale ranging from worst state of health imaginable to best state of health imaginable.

Rational Experiential Inventory, Short Form 10:

The Rational Experiential Inventory is a psychometric instrument designed to evaluate individual differences in intuitive-experiential and analytical-rational thinking, which has been validated in diverse populations. A short, 10-item form has been found to have similar properties as the original version[18-19].

Hypothetical questions regarding life-sustaining interventions:
The hypothetical scenarios and accompanying questions are based on common clinical scenarios that patients face and include decisions about surgical feeding tubes, dialysis, antibiotics in the setting of terminal illness and mechanical ventilation. They are similar to hypotheticals presented in prior studies of patient preferences regarding life-sustaining treatment[16, 20].

Decisional Conflict Scale, Uncertainty Subscale:
The Decisional Conflict Scale[21] is a well-validated instrument used to assess patients certainty in making healthcare decisions. The psychometric properties of the uncertainty subscale used independently have been demonstrated in a study of tools to evaluate shared decision making[22].

Spielberger State-trait Anxiety Scale, Short Form:
The Spielberger State-Trait Anxiety Inventory is a validated instrument to look at the presence of anxiety over a preceding period of time (when asked in the trait form) and in a particular moment (when asked in the state form)[23]. The short form has been shown to deliver results similar to the full inventory[24]. We will use the wording for the state form of the scale to evaluate how anxious the patient feels after answering the questions regarding life-sustaining treatment.

Questions to assess analytical reasoning:
During pilot testing of our methods, we will use a variety of questions to assess the analytical processing of the participants, all of which have been used repeatedly by researchers in cognitive psychology as measures of analytical thinking. These include denominator neglect problems[25], belief bias syllogisms[26-27], and the Cognitive Reflection Test[28].

1.5 Subject recruitment

We will recruit patients with respiratory, cardiovascular and oncological diseases from the population of stable inpatients on the general wards at the Hospital of the University of Pennsylvania. Each day, the research coordinator will screen the electronic medical records of patients on the wards (rotating which wards are screened on which day) to determine their eligibility using the eligibility criteria outlined above. The research coordinator will contact each attending physician on the services from which patients will be recruited when the attending physician comes on service to inform the attending of the research project and get the attending physicians' agreement regarding patient recruitment. If the physician does not respond, then the research coordinator will contact the physician once eligible patients have been identified to 1) alert them to their patients eligibility for participation 2) inform them their patients will be recruited for enrollment; and 3) provide them an opportunity to decline or defer any given patients enrollment. Research coordinators will approach potential study participants while they are in their hospital rooms.

1.6 Subject compensation

Subjects will not receive financial compensation for participation in this study.

1.7 Study Procedures

1.7.1 Consent

Eligible patients will be approached by a research coordinator for consent while they are in their hospital rooms. Research coordinators will seek patients consent to participate in a study comparing different methods of decision making about life-sustaining treatment. The research coordinator will specify that the questions posed in the study regarding preferences for life-sustaining treatments are hypothetical, but that we would like the patients to approach the questions as if they are answering them in a true clinical context. Following discussion of the study, research coordinators will obtain written consent from patients. The consent forms will contain HIPAA statements of authorization of release of medical records, thus facilitating our collection of data from medical records during the study. The consent form will indicate that patients are being invited to participate in the study because they have one of a series of medical diagnoses.

1.7.2 Procedures

The research coordinators will screen electronic medical records of inpatients on the general medicine, pulmonary, cardiology and oncology wards at the Hospital of the University of Pennsylvania for eligibility. Patients' eligibility status will be entered into the eligibility database. We will record ICD9 and ICD 10 codes. Eligible patients will be approached by a research coordinator in the inpatient setting who will seek patients consent to participate in a study exploring patient decision making about life-sustaining medical treatments. The research coordinator will specify that the questions are hypothetical and that the answers are not binding, but emphasize that we would like patients to answer the questions as if they were real. Following discussion of the study, research coordinators will obtain written consent from patients. The consent forms will contain HIPAA statements of authorization of release of medical records, thus facilitating our collection of data from medical and billing records during the study. The consent includes clear explanations that different types of decision making will be assigned by chance. The research coordinators will explain who will be enrolled, how many patients are being targeted for enrollment, and what the outcomes of interest are.

Pilot Testing: During pilot testing, we will test several methods for encouraging patients in the intuitive arm to answer the questions based on intuition including: (a) a time constraint condition, under which patients will be asked to make a decision about the four hypothetical questions based on gut instinct and to answer each question within one minute; and (b) a cognitive loading condition, under which patients will be asked to attend to a second task while also reading the hypothetical scenarios and answering the questions about whether they would or would not want the therapy in question. The second task will either be retention in working memory of a string of numbers or a task that involves pressing a button every time a specific number appears on the bottom of the computer screen. We will ask patients in each condition how difficult the task was to verify that the cognitive load group found the task significantly harder than the other groups. We will also administer a test of analytical reasoning to groups in the various pilot arms to establish that time constraint and cognitive load result in lower ability to engage in analytical reasoning that the group without a time constraint or a cognitive load. The tests we will use to assess analytical reasoning include denominator neglect questions[25], belief bias syllogisms[26] and the Cognitive Reflection Test[28]. We will also pilot test doing the interviews for both arms in a single survey session and in two separate sessions separated by as much as 24 hours. Once we determine during pilot testing the logistics of how we will organize the intuition and deliberation arms, we will submit a modification describing the final proposed technique.

The questions will be administered as a Qualtrics survey, facilitated by the research coordinator. At this point, we anticipate that patients in each arm will have two facilitated survey sessions with the research coordinator, as follows (although we may do a single session for one or both arms depending on the results of the pilot testing).

Intuitive arm: After patient consent is obtained, the research coordinator will administer the hypothetical questions regarding medical interventions, along with the Decision Conflict Scale uncertainty subscale and the short form of the state-trait anxiety scale. At a second visit on the same day, the research coordinator will administer the demographics survey, the questions about health state valuation, and the Rational Experiential Inventory.

Deliberative arm: After patient consent is obtained, the research coordinator will give the patient instructions and then give the patient a hard copy of the hypothetical questions regarding specific medical interventions. The patient will be given instructions to deliberate on the questions until the following day. At a second visit the following day, the research coordinator will return and collect the patient's answers to the intervention questions. The research coordinator will then administer the Decision Conflict Scale uncertainty subscale, the short form of the state-trait anxiety scale, the demographics survey, the questions about health state valuation, and the Rational Experiential Inventory.

1.7.3 Administration of surveys and/or process

The research coordinator will screen electronic medical records of inpatients on the general medicine, pulmonary, cardiology and oncology wards at the Hospital of the University of Pennsylvania for eligibility. Patients' eligibility status will be entered into the eligibility database. We will record ICD9 and ICD 10 codes. The research coordinator will contact each attending physician on the services from which patients will be recruited when the attending physician comes on service to inform the attending of the research project and get the attending physician's agreement regarding patient recruitment. If the physician does not respond, then the research coordinator will contact the physician once eligible patients have been identified to 1) alert them to their patients eligibility for participation 2) inform them their patients will be recruited for enrollment; and 3) provide them an opportunity to decline or defer any given patients enrollment. Eligible patients will be approached by a research coordinator in the inpatient setting. The research coordinator will seek patients consent to participate in a study exploring patient decision making about life-sustaining medical treatments. The research coordinator will specify that the questions are hypothetical and that the answers are not binding, but emphasize that we would like patients to take the questions seriously and give their best answer as if they were being asked in a clinical context. Following discussion of the study, the research coordinator will obtain written consent from the patient. The consent forms will contain HIPAA statements of authorization of release of medical records, thus facilitating our collection of data from medical records during the study. The questions will be administered as a Qualtrics survey, facilitated by the research coordinator. Patients in each arm most likely will have two facilitated survey sessions with the research coordinator, as follows (we will be making some adjustments based on results of pilot testing, described in the Study Procedures section; it is possible that subjects in the intuitive arm will have only one survey session).

Intuitive arm: After patient consent is obtained, the research coordinator will administer the hypothetical questions regarding medical interventions, along with the Decision Conflict Scale uncertainty subscale and the short form of the state-trait anxiety scale. At a second visit on the same day, the research coordinator will administer the demographics survey, the questions about health state valuation, and the Rational Experiential Inventory.

Deliberative arm: After patient consent is obtained, the research coordinator will give the patient instructions and then give the patient a hard copy of the hypothetical questions regarding specific medical interventions. The patient will be given instructions to deliberate on the questions until the following day. At a second visit the following day, the research coordinator will return and collect the patient's answers to the intervention questions. The research coordinator will then administer the Decision Conflict Scale uncertainty subscale, the short form of the state-trait anxiety scale, the demographics survey, the questions about health state valuation, and the Rational Experiential Inventory.

We anticipate that the total time for the two sessions spent administering the survey with each patient will not exceed forty minutes. Subject IDs will be assigned at the point of consent. Subject ID numbers, demographic information, medical record number and group assignments will be entered into the analytic database and into Qualtrics. We will maintain the link between the patient name, medical record number and subject ID only until the interviews with that patient are complete and then the names and medical record numbers will be deleted.

1.8 Human research protection

1.8.1 Data management

Prudent steps will be taken to ensure that all information will be kept confidential and secure, including medical and survey data. When a subject consents, he will be assigned a unique randomly generated study identifier. That unique identifier will be entered into every survey/database that is used. A separate password-protected file linking the patient to his unique identifier will be maintained on a secure server only until the final interview is complete. Then the link will be destroyed. Participants answers to questions will be entered in a secured database maintained by Qualtrics. Qualtrics uses Transport Layer Security (TLS) encryption (also known as HTTPS) for all transmitted data. They also protect surveys with passwords and HTTP referrer checking. Their data is hosted by third party data centers that are SSAE-16 SOC II certified. All data at rest are encrypted, and data on deprecated hard drives are destroyed by U.S. DOD methods and delivered to a third-party data destruction service. Qualtrics deploys the general requirements set forth by many Federal Acts including the FISMA Act of 2002. They meet or exceed the minimum requirements as outlined in FIPS Publication 200.

1.8.2 Subject confidentiality

Steps will be taken to ensure that all information will be kept confidential and secure. Unique patient identifiers numbers will be assigned to each subject locally and kept in a secure encrypted file. All paper records will be kept in locked files; all computers will be password protected and kept in locked rooms; all databases will be password protected and maintained on encrypted hard-drives; none will be stored on stand-alone PCs or laptops. The only link between patient name and unique identifier code will be destroyed as soon as the interviews with that patient are complete.

1.9 Risk/Benefit

1.9.1 Potential study risks

The potential risks to human subjects in this research include (1) risks of breach of confidentiality of personal health information (PHI); and (2) risks of emotional distress brought on by being asked to contemplate end-of-life care.

1.9.2 Potential study benefits

The primary benefits to be gained are those related to the general knowledge to be obtained from the study about how patients with chronic illnesses think about life-sustaining medical interventions. Participants in this study may benefit directly from the opportunities to consider their end-of-life care preferences.

1.9.3 Risk/benefit assessment

This study presents no more than minimal risk. Many precautions will be taken to protect subjects against the most likely risk, which is breach of confidentiality. In addition, the answers to the questions posed about medical interventions are not binding nor will expressed preferences be communicated to the health care providers taking care of the patients enrolled in the study. Therefore, the answers to the questions are unlikely to erect barriers to patients receiving desired care. We believe the benefits to individual subjects in terms of learning about decisions regarding life-sustaining medical interventions will outweigh any potential risk.

1.10 Summary of changes to protocol

1. Expanded the eligibility criteria to include patients 60 years of age with earlier stages of cardiac, pulmonary or oncologic diseases if they had at least one prior hospitalization during the past year.

2. After the first 20 patients were enrolled, we added a question about general life satisfaction, a question about perceived life expectancy, a question about extent of prior deliberation on end-of-life care, a question about preference for general approach to treatment in the event of serious illness, and a modified time tradeoff question designed to understand whether patients would trade time alive to avoid ICU care at the end of life

3. Obtained a waiver of consent for collection of information regarding whether there is a living will in the electronic medical record for all patients approached to participate in the study, including those who do not consent. For all patients approached, including both those who do and do not consent, we collected information from the electronic health record for the University of Pennsylvania Health System regarding whether there is a living will for that patient on file.

4. We relaxed the requirement for a second visit to obtain the health state evaluations to facilitate complete data capture. Patients in the deliberative arm were still required to wait for at least 1 minute prior to making health care choices, but in both arms we allowed complete data capture to occur during the same visit.

2 Statistical analytic plan

The primary outcome will be the choices patients make whether to accept or decline the life-sustaining interventions. The primary exposure will be whether participants were responding in the intuition or the deliberation arm. Chi-square testing will be used to compare these arms. In secondary analyses, we will evaluate congruence between stated valuations of health states and treatment acceptance or refusal, which will be intended as a proxy for decision quality. We will use a logistic regression model to determine how valuations of relevant health states influence the refusal or acceptance of treatments; the resulting odds ratio will represent the incremental odds of refusing treatment with one unit increase on the valuation scale. Because we are interested in the relationship between the scale and treatment refusal across groups defined by randomization, we will additionally explore an interaction between scale and randomization group. Finally, we will analyze the difference between the means of the two randomization groups on the state anxiety scale and the uncertainty subscale of the Decisional Conflict Scale. These data will be described using means and t-tests if normally distributed or medians and Wilcoxon rank sum tests if not normally distributed.

2.1 Summary of changes to statistical analytic plan

1. After the enrollment of the first 20 subjects, but prior to evaluating any data, we specified two effect modification analyses reflecting our additions of information about prior living wills or goals of care conversations. Specifically, we evaluated statistical interactions between either of these exposures and the intervention on the primary outcomes of the end-of-life choices.
2. After reviewing the results, we decided to calculate Bayes factors to provide additional information, above and beyond the p values, to aid in interpretation of our results.

3 References

1. Stanovich, K. E., & West, R. F. (2000). Individual differences in reasoning: Implications for the rationality debate. Behavioral and Brain Sciences, 23, 645665.
2. Kahneman, D. (2003). A perspective on judgment and choice: Mapping bounded rationality. The American Psychologist, 58(9), 697720.
3. Kahneman, Daniel, and Shane Frederick. "Frames and brains: elicitation and control of response tendencies." Trends in cognitive sciences 11.2 (2007): 45-46.
4. Tversky, Amos, and Daniel Kahneman. "Judgment under uncertainty: Heuristics and biases." science 185.4157 (1974): 1124-1131.
5. Dijksterhuis, Ap, et al. "On making the right choice: The deliberation-without-attention effect." Science 311.5763 (2006): 1005-1007.
6. Usher, Marius, et al. "The impact of the mode of thought in complex decisions: Intuitive decisions are better." Frontiers in psychology 2 (2011).
7. Greene, Joshua D., et al. "Cognitive load selectively interferes with utilitarian moral judgment." Cognition 107.3 (2008): 1144-1154.
8. Suter, Renata S., and Ralph Hertwig. "Time and moral judgment." Cognition 119.3 (2011): 454-458.
9. Gervais, Will M., and Ara Norenzayan. "Analytic thinking promotes religious disbelief." Science 336.6080 (2012): 493-496.
10. Rand, D. G., Greene, J. D., & Nowak, M. a. (2012). Spontaneous giving and calculated greed. Nature, 489(7416), 42730. doi:10.1038/ nature11467
11. Rand, David G., and Ziv G. Epstein. "Risking Your Life Without a Second Thought: Intuitive Decision-Making and Extreme Altruism." Available at SSRN 2424036 (2014).
12. Stacey, Dawn, et al. "Decision aids for people facing health treatment or screening decisions." Cochrane Database Syst Rev 10.10 (2011).
13. Fried, Terri R., et al. "Understanding the treatment preferences of seriously ill patients." New England Journal of Medicine 346.14 (2002): 1061-1066.
14. Silveira, Maria J., Scott YH Kim, and Kenneth M. Langa. "Advance directives and outcomes of surrogate decision making before death." New England Journal of Medicine 362.13 (2010): 1211-1218.
15. Connors, Alfred F., et al. "A controlled trial to improve care for seriously iII hospitalized patients: The study to understand prognoses and preferences for outcomes and risks of treatments (SUPPORT)." Jama 274.20 (1995): 1591-1598.
16. Patrick, Donald L., et al. "Validation of preferences for life-sustaining treatment: implications for advance care planning." Annals of Internal Medicine 127.7 (1997): 509-517.
17. Kopec, Jacek A., and Kevin D. Willison. "A comparative review of four preference-weighted measures of health-related quality of life." Journal of clinical epidemiology 56.4 (2003): 317-325.
18. Epstein, Seymour, et al. "Individual differences in intuitiveexperiential and analyticalrational thinking styles." Journal of personality and social psychology71.2 (1996): 390.
19. Pacini, Rosemary, and Seymour Epstein. "The relation of rational and experiential information processing styles to personality, basic beliefs, and the ratio-bias phenomenon." Journal of personality and social psychology 76.6 (1999): 972.
20. Fried, Terri R., et al. "Understanding the treatment preferences of seriously ill patients." New England Journal of Medicine 346.14 (2002): 1061-1066.
21. O'Connor, Annette M. "Validation of a decisional conflict scale." Medical decision making 15.1 (1995): 25-30.
22. Légaré, France, et al. "Some but not all dyadic measures in shared decision making research have satisfactory psychometric properties." Journal of clinical epidemiology 65.12 (2012): 1310-1320.

23. Spielberger, Charles D. StateTrait Anxiety Inventory. John Wiley & Sons, Inc., 2010.
24. Marteau, Theresa M., and Hilary Bekker. "The development of a sixitem shortform of the state scale of the Spielberger StateTrait Anxiety Inventory (STAI)."British Journal of Clinical Psychology 31.3 (1992): 301-306.
25. Kirkpatrick, Lee A., and Seymour Epstein. "Cognitive-experiential selftheory and subjective probability: further evidence for two conceptual systems. Journal of personality and social psychology 63.4 (1992): 534.
26. Evans, J. St BT, Julie L. Barston, and Paul Pollard. "On the conflict between logic and belief in syllogistic reasoning." Memory & cognition 11.3 (1983): 295-306.
27. Markovits, Henry, and Guilaine Nantel. "The belief-bias effect in the production and evaluation of logical conclusions." Memory & Cognition 17.1 (1989): 11-17.
28. Frederick, Shane. "Cognitive reflection and decision making." Journal of Economic perspectives (2005): 25-42.
