# Supplementary material for: Intuitive vs Deliberative Approaches to Making Decisions About Life Support: A Randomized Clinical Trial
Source: JAMA Netw Open. 2019 Jan 25;2(1):e187851. doi: 10.1001/jamanetworkopen.2018.7851 (PMC6484534; doi:10.1001/jamanetworkopen.2018.7851)
Supplement: Supplement 2. — eFigure 1. Study Inclusion and Exclusion Criteria eFigure 2. Distribution of Primary Diagnoses Among Patients Enrolled in the Study eFigure 3. Scenarios Requiring Decision About Life-Sustaining Treatment eFigure 4. Distribution of Subjective Rating of Mental Effort in Intuitive Arm Versus Deliberative Arm eTable 1. Interaction Between Selected Characteristics and Treatment Arm for the Outcome of Treatment Decisions eTable 2. Decisional Uncertainty by Arm [file jamanetwopen-2-e187851-s002.pdf]

Supplementary Online Content

Rubin EB, Buehler AE, Cooney E, Gabler NB, Mante AA, Halpern SD. Intuitive vs deliberative approaches to making decisions about life support: a randomized clinical trial. *JAMA Netw Open*. 2019;2(1):e187851. doi:10.1001/jamanetworkopen.2018.7851

eFigure 1. Study Inclusion and Exclusion Criteria
eFigure 2. Distribution of Primary Diagnoses Among Patients Enrolled in the Study
eFigure 3. Scenarios Requiring Decision About Life-Sustaining Treatment
eFigure 4. Distribution of Subjective Rating of Mental Effort in Intuitive Arm Versus Deliberative Arm
eTable 1. Interaction Between Selected Characteristics and Treatment Arm for the Outcome of Treatment Decisions
eTable 2. Decisional Uncertainty by Arm

This supplementary material has been provided by the authors to give readers additional information about their work.

© 2019 Rubin EB et al. *JAMA Network Open.*

eFigure 1. Study inclusion and exclusion criteria

Inclusion criteria:

1. Age 60 or older
2. Speaks and reads fluently in English
3. Currently an inpatient at the Hospital of the University of Pennsylvania
4. Stable vital signs
5. Listed as “full code” in the electronic medical record
6. Currently admitted to the oncology, cardiology or pulmonary service and has been admitted to the same service at least once in the last year; and/or has one or more of the following diagnoses:

- Chronic obstructive pulmonary disease with at least severe airflow obstruction on most recent spirometry and/or eligible for long-term oxygen therapy
- Incurable interstitial lung disease with at least severe restriction on most recent pulmonary function tests and/or eligible for long-term oxygen therapy
- Congestive heart failure with NYHA Class III or higher and current hospitalization related to heart failure
- Acute myeloid leukemia
- Stage IV lymphoma
- Stage IIIB or Stage IV non-small cell lung cancer, cholangiocarcinoma, renal cell carcinoma, breast cancer, uterine cancer, cervical cancer, ovarian cancer, colorectal cancer, gastric cancer, pancreatic cancer, prostate cancer, urothelial cancer
- Stage C or D hepatocellular carcinoma
- Mesothelioma or any malignancy metastatic to the pleura

Exclusion criteria

- Cognitive impairment to the point where patient is unable to provide informed consent or to understand instructions
- Uncontrolled or poorly controlled pain, dyspnea or other symptoms on the day of recruitment
- Currently has feeding tube or tracheostomy tube
- Currently undergoing evaluation for solid organ transplantation
- First admission to the hospital following diagnosis of a serious illness

© 2019 Rubin EB et al. *JAMA Network Open.*

eFigure 2. Distribution of primary diagnoses among patients enrolled in the study

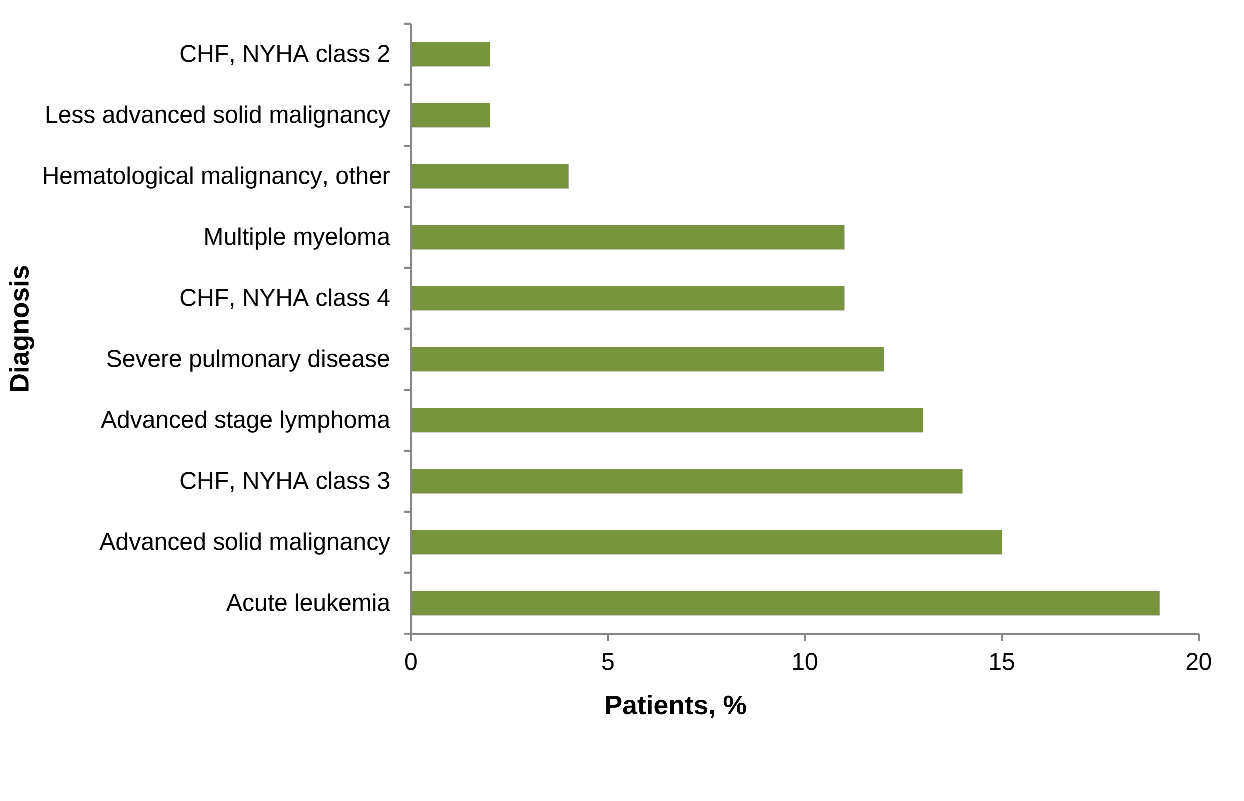

Abbreviations*:* NYHA, New York Heart Association
"Advanced solid malignancy" was defined as Stage IIIB or Stage IV non-small cell lung cancer, cholangiocarcinoma, renal cell carcinoma, breast cancer, uterine cancer, cervical cancer, ovarian cancer, colorectal cancer, gastric cancer, pancreatic cancer, prostate cancer, urothelial cancer, stage C or D hepatocellular carcinoma, mesothelioma or any malignancy metastatic to the pleura. Lymphoma patients all had stage IV disease.

© 2019 Rubin EB et al. *JAMA Network Open.*

eFigure 3. Scenarios requiring decision about life-sustaining treatment

Imagine that in the future you become very weak and develop difficulty swallowing. You are unable to eat or drink safely. When you do, small amounts of food and liquid go into your lungs and cause trouble with your breathing. Doctors think that this will be a permanent condition. The only way for you to get nutrition safely is to have a feeding tube inserted into your stomach. For the rest of your life, you will most likely have some trouble getting around, need some help with basic life activities like dressing yourself, and have mild pain or discomfort. Under these circumstances, would you choose:

- To have a feeding tube inserted into your stomach to give you food and fluids with the goal of living as long as possible; or
- Not to have a feeding tube inserted into your stomach and continue to eat and drink small amounts, understanding that you might die sooner than you would if you had a feeding tube inserted

---

Imagine that in the future you become ill to the point that you are expected to die within the next several months. This means that you have some days when you drift in and out of awareness, have some discomfort that requires medication, are in bed most of the time due to weakness and need help getting dressed, bathing, and using the bathroom. You develop an infection. If you receive antibiotics, you might live another several months in a nursing home or similar facility in the condition I described above. If you don't receive antibiotics, you likely will die within a week to two weeks. Under these circumstances, would you choose:

- To treat the infection with the goal of living as long as possible; or
- Not to treat the infection, receive medication to treat any uncomfortable symptoms, and die at home

---

Imagine that in the future you develop a life-threatening illness with difficulty breathing. Doctors think there is a 50% chance you will survive this illness. In order to survive, you will likely need support from a breathing machine for at least two weeks. Doctors expect that if you survive and go through a long period of rehabilitation, you will be able to return home. If you survive, it is likely that you will have a lot of trouble getting around, will need some help with activities like getting dressed and using the bathroom, and will have mild pain or discomfort. Under these circumstances, would you choose:

- To have the breathing tube put in; or
- To be made comfortable with medications and die without a breathing tube

---

Imagine that you were to become very sick in the near future and your loved ones and doctors were trying to decide how to best care for you. I am going to describe three general approaches to medical treatment in the event of serious illness. Please tell me which approach best describes how you would wish to be treated if you were to become very sick in the near future.

- If I were to become very sick, I would want the main focus of my care to be on treating any uncomfortable symptoms I might have and not on keeping me alive as long as possible. I would want to limit the amount of time I spent in the hospital and the number of tests and treatments I received. I would not wish to have any procedures that would cause me pain and I would not want to go to the intensive care unit. I would want life support measures only if they were absolutely necessary to keep me comfortable and would not want to be put or kept on life support with the goal of extending my life. I would like to be allowed to die naturally. If my heart were to stop, I would not want my medical team to take extraordinary measures to try to restart it.

© 2019 Rubin EB et al. *JAMA Network Open.*

- If I were to become very sick, I would want the main focus of my care at first to be on keeping me alive as long as possible, even if that means that I would have to spend a lot of time in the hospital and have some procedures done that might cause me pain or discomfort. If I needed to go to the intensive care unit, I would want to do that. If I needed life support measures to keep me alive, I would want those for a little while. But I would not want to live for a long time on life support or in another condition where I would be unable to leave the hospital or another health care facility. If after a trial of life support, it looked very unlikely that I would get better and be able to go home, I would want the main focus of my care to shift to treating any uncomfortable symptoms I have and away from trying to keep me alive as long as possible. I would like to be allowed to die naturally. If my heart were to stop, I would not want my medical team to take extraordinary measures to try to restart it.

- If I were to become very sick, I would want the focus of my care to be on keeping me alive as long as possible, even if that means that I would have to spend a lot of time in the hospital and have some procedures done that might cause me pain or discomfort. If I needed to go to the intensive care unit, I would want to do that. If I needed life support measures to keep me alive, I would want to have them. I would want to have any medical treatment or procedure that would keep me alive. If my heart were to stop, I would want my medical team to take every possible measure to try to restart it.

The figure depicts the distribution in the two arms of subjective ratings of mental effort required to remember the designated numbers while hearing the scenarios and answering questions about preferences (0 = very, very little; 10 = very, very much). 93% of patients in the deliberative arm rated the mental effort as 0, while 72% of patients in the intuitive arm rated the mental effort as 5 or greater. n=97 in the intuitive arm; n=102 in the deliberative arm.

© 2019 Rubin EB et al. *JAMA Network Open.*

eFigure 4. Distribution of subjective rating of mental effort in intuitive arm versus deliberative arm

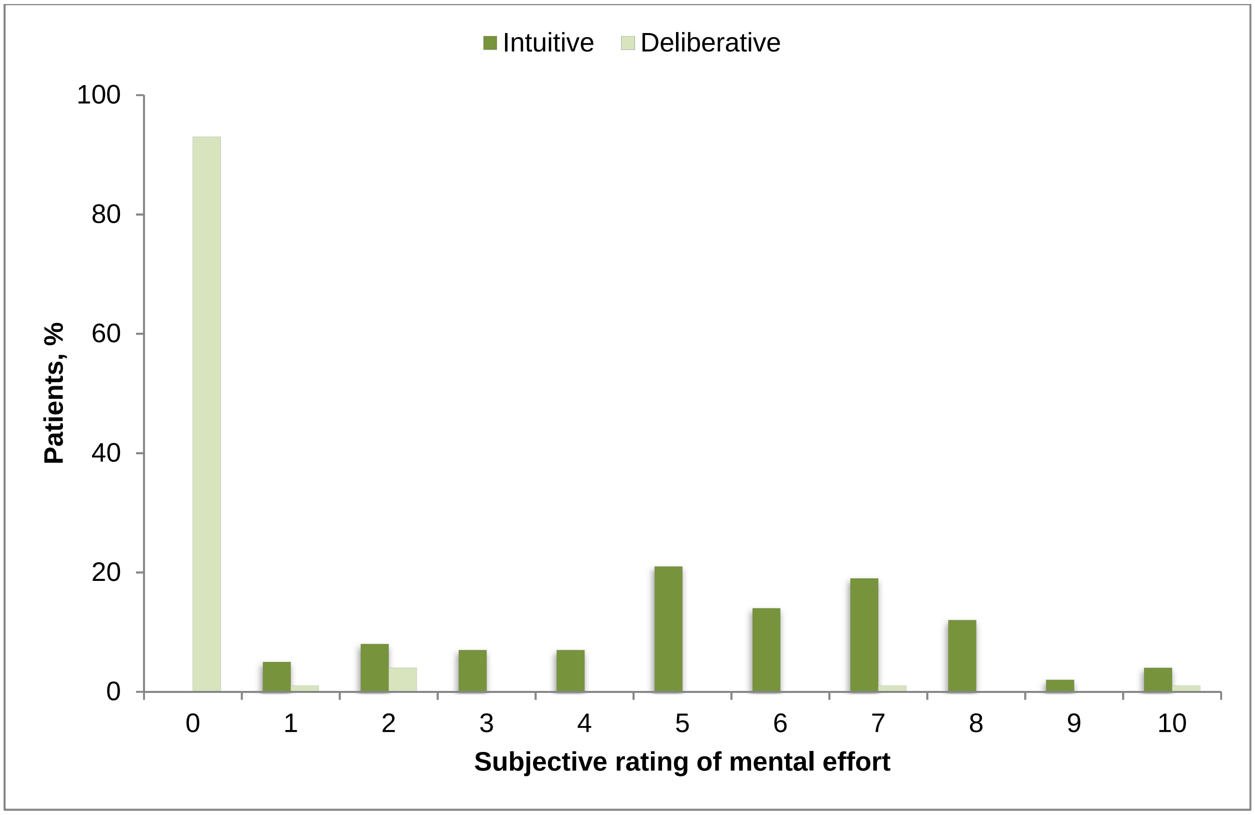

	0	1	2	3	4	5	6	7	8	9	10
Deliberative	93%	4%	1%	0%	0%	0%	0%	1%	0%	0%	1%
Intuitive	0%	5%	8%	7%	7%	21%	14%	19%	12%	2%	4%

eTable 1. Interaction between selected characteristics[a] and treatment arm for the outcome of treatment decisions

	Patients, (%)		
	Intuitive Arm	Deliberative Arm	*P* value of interaction term
Accept feeding tube			.47
Living will yes[b]	32	38	
Living will no or don’t know	56	52	
Accept antibiotics			.10
Living will yes	24	38	
Living will no or don’t know	58	52	
Accept intubation			.53
Living will yes	52	57	
Living will no or don’t know	67	64	
Accept tracheostomy			.67
Living will yes	36	36	
Living will no or don’t know	38	46	
Preference for high intensity treatment			.53
Low intensity			
Living will yes	35	53	
Living will no or don’t know	24	34	
Moderate intensity			
Living will yes	55	38	
Living will no or don’t know	37	50	
High intensity			
Living will yes	10	9	
Living will no or don’t know	40	16	
Accept feeding tube			.03
Previous EOL discussions yes	47	36	
Previous EOL discussions no	36	56	
Accept antibiotics			.94
Previous EOL discussions yes	38	43	
Previous EOL discussions no	41	44	
Accept intubation			.97
Previous EOL discussions yes	56	57	
Previous EOL discussions no	62	27	
Accept tracheostomy			.26
Previous EOL discussions yes	39	51	
Previous EOL discussions no	35	27	
Preference for high intensity treatment			.34
Low intensity			
Previous EOL discussions yes	31	43	
Previous EOL discussions no	28	49	
Moderate intensity			
Previous EOL discussions yes	49	43	
Previous EOL discussions n0	44	42	
High intensity			

© 2019 Rubin EB et al. *JAMA Network Open.*

Previous EOL discussions yes	20	13	
Previous EOL discussions no	28	9	

Abbreviation: EOL, end of life.
[a]This table reports treatment decisions by arm stratified by (a) endorsement of living will and (b) endorsement of having had at least two conversations in the last year about end of life treatment preferences. The decisions are reported as percentages.
[b]Sample sizes: Living will yes, n=54 in intuitive arm, n=58 in deliberative arm; Living will no or don't know, n=43 in intuitive arm, n=44 in deliberative arm; previous EOL discussions yes, n=55 in intuitive arm, n=61 in deliberative arm; previous EOL discussions no, n=42 in intuitive arm, n=41 in deliberative arm

© 2019 Rubin EB et al. *JAMA Network Open.*

eTable 2. Decisional uncertainty[a] by arm

Scenario	Mean (SD)			*P* value
	Total Sample (n=199)	Intuitive Arm (n=97)	Deliberative Arm (n=102)	
Feeding tube	21 (22)	21 (23)	21 (21)	.86
Antibiotics	20 (21)	20 (22)	19 (21)	.65
Intubation	23 (24)	26 (26)	21 (22)	.11
Tracheostomy	26 (25)	29 (25)	24 (25)	.22
Treatment approach	21 (22)	23 (22)	20 (21)	.33

[a]Decisional uncertainty was evaluated using the uncertainty subscale of the Decisional Conflict Scale. 0 = feels extremely certain about best choice, 100 = feels extremely uncertain about best choice. All values are reported as mean (standard deviation).

© 2019 Rubin EB et al. *JAMA Network Open.*
