# Supplementary material for: Intuitive vs Deliberative Approaches to Making Decisions About Life Support: A Randomized Clinical Trial
Source: JAMA Netw Open. 2019 Jan 25;2(1):e187851. doi: 10.1001/jamanetworkopen.2018.7851 (PMC6484534; doi:10.1001/jamanetworkopen.2018.7851)
Supplement: Supplement 3. — Data Sharing Statement [file jamanetwopen-2-e187851-s003.pdf]

Data Sharing Statement

Rubin. Intuitive vs Deliberative Approaches to Making Decisions About Life Support. *JAMA Netw Open*. Published January 25, 2019. 10.1001/jamanetworkopen.2018.7851

Data
Data available: Yes
Data types: Deidentified participant data, Data dictionary
How to access data: rubin3@partners.org
When available: With publication

Supporting Documents
Document types: Informed consent form
How to access documents: rubin3@partners.org
When available: With publication

Additional Information
Who can access the data: researchers whose proposed use of the data has been approved
Types of analyses: for any approved purposes
Mechanisms of data availability: after approval of a proposal
